# An Improved Low-Noise Processing Methodology Combined with PCL for Industry Inspection Based on Laser Line Scanner

**DOI:** 10.3390/s19153398

**Published:** 2019-08-02

**Authors:** Jianxiong Li, Qian Zhou, Xinghui Li, Ruiming Chen, Kai Ni

**Affiliations:** 1 Graduate School at Shenzhen, Tsinghua University, Shenzhen 518055, China; 2 Tsinghua Shenzhen International Graduate School, Tsinghua University, Shenzhen 518055, China

**Keywords:** 3D reconstruction, laser line scanner, industry inspection, registration algorithm

## Abstract

This paper introduces a three-dimensional (3D) point cloud data obtained method based on a laser line scanner and data processing technology via a PCL open project. This paper also provides a systematical analysis of the error types of laser line scanner and common error reducing solutions and calibration of the laser line scanner. The laser line scanner is combined with a precision motorized stage to obtain the 3D information of a measurand, and the format of point cloud data is converted via the set of *x*, *y*, and *z* coordinates. The original signal is processed according to the noise signal types of the raw point cloud data. This paper introduced a denoise process step by step combining various segmentation methods and a more optimized three-dimensional data model is obtained. A novel method for industry inspection based on the numerous point cloud for the dimensions evaluation via feature extraction and the deviation of complex surface between scanned point cloud and designed point cloud via registration algorithm is proposed. Measurement results demonstrate the good performance of the proposed methods. An obtained point cloud precision of ±10 μm is achieved, and the precision of dimension evaluation is less than ±40 μm. The results shown in the research demonstrated that the proposed method allows a higher precision and relative efficiency in measurement of dimensions and deviation of complex surfaces in industrial inspection.

## 1. Introduction

Three-dimensional (3D) reconstruction is an important process in industrial production. Namely, it is used to obtain the 3D contour information of measured objects. At present, it is a research hotspot and has wide application prospects in the future. The main task of 3D reconstruction is acquisition and processing of 3D point cloud data. The methods for obtaining 3D point cloud data, according to the scale and feature size of the measured object, include the micro- or nano-scales, industrial application level, and large measurement range scale. Chromatic confocal sensor, atomic force microscopy (AFM), and scanning electron microscope (SEM), etc., can be used for 3D reconstruction in micro- or nano-scales. Depth camera, coordinate measuring machine (CMM), and laser line scanner (LLS) denote the acquisition methods for the industrial manufacturing industry, and lidar can be used as the means of building reconstruction for large scale measurement [1,2,3]. In the SEM and the other 3D modeling for micro and nano-fabrication to obtain the micro-morphology of the measured object, the measurement range is generally narrow. Generally, it is between tens and hundreds of micrometers [4]. The CMM has relatively high precision and a larger measurement range. However, the CMM faces a big challenge when rapid on-line measurement is required because of its low efficiency [5]. Lidar has a wide measurement range of up to several hundred meters, but the measurement precision can reach only the cm-level [6]. A depth camera has a slightly smaller measurement range of a few hundreds of millimeters, and the measurement precision is on the mm-level [7]. The LLS measurement range is comparable to that of a depth camera, while its measurement precision can reach several micrometers and has a rapid data acquisition, which is suitable for relatively high-precision inspection, although it has a big challenge in the data acquisition, which always involves several noises [5,8]. Compared with the CMM, lidar, and depth camera, the LLS has more probability to be employed in online inspection of industrial manufacturing if the data processing techniques can be improved.

Aiming to facilitate 3D construction technique applications based on the LLS into online inspection of industrial manufacturing, the data acquisition and pre-processing of the raw data by denoising is of the first importance. When the original data is acquired using the LLS, the noise points inevitably exist in a 3D point cloud, and their existence has a great influence on data processing, such as model evaluation and registration. In order to solve this problem, in 2008, Rusu et al. proposed a filtering method based on the threshold segmentation for single dimension data and a statistical method to remove the outliers in the point cloud data, which could remove the scanning occlusion points to a certain degree [9]. This method could also improve the accuracy of 3D data acquisition. However, due to the inherent limitations of the statistical methods used, the precision of removing noise points was largely related to the confidence interval of statistical results, which often caused a misjudgment when noise points were removed. In 2012, Budak et al. proposed use of segmentation in the 3D point cloud to extract valuable information to divide a contour with a planar model evaluation or other regular models such as cylinders and spheres. The parameters of the evaluated model could be obtained using set threshold factors [10]. The advantage of this method is that it has a good removal effect of noise points close to the model, such as edge noise points, which cannot be removed by the statistical methods. However, as for the noise points in the selected sub-model, such as the selected planar model, the noise points appearing in the plane still exist, which severely limits the application of 3D reconstruction technology in industrial manufacturing, which further hinders the efficiency and quality of industrial production to some extent. Moreover, they state the limitation of their processing is time consuming. It can be roughly estimated at about 350,000 points per hour [10]. In sum, these methods can remove noise from the point cloud to some extent, but some noise will still exist after the above method employed in pre-processing, especially for metallic parts.

What is more, for functionalizing the LLS based 3D reconstruction, including geometry measurement, surface evaluation, and so forth, an applicable data processing system in necessary. At the beginning, the point cloud processing technology was proposed by Rusu in 2011 using a depth camera to obtain the 3D information and to perform the corresponding data processing, mainly for applications in household environments [11]. Schleich et al. studied the theoretical calculation methods of surface topography tolerance for some random point sets based on computer technology and highlighted the advantages of these methods in industrial inspection applications [12]. In [13], Kumar introduced the application case of point cloud obtained by the CMM and LLS for gear reconstruction and processing by modern digitizing technique and advanced CAD systems. However, the difference of placing position influences the point cloud precision comparing with gear before wear. Albasset and Thomas obtained point cloud data via lidar and studied use of 3D point cloud registration methods for quality testing for architectural research [14]. In sum, because these abovementioned methods are limited in both efficiency and precision, they are hardly employed for automatically online dimension and geometry inspections of mechanical parts where high precision, efficiency, and robustness are highly required.

In order to solve all mentioned problems, we proposed several new methods. First of all, the sources of noise points are categorized into three types according to distance between the noise points and the object. These three types of noise points are then removed by designing different filters. For the points away from the object, a statistical filter is capable of removing it. Based on Budak’s method [10], a sub-model evaluation and denoising combined with the *k*-nearest neighbor (KNN) search algorithm is used, which greatly improves the efficiency of noise removal. Finally, a region growing algorithm, which uses the local connectivity and surface smoothness as the two constraints, is used to limit the connected domain. The discrete points that still exist in the sub-model can be removed, and finally, a high signal-to-noise ratio (SNR) of the entire model is obtained. With the denoised 3D information of the object, the tolerance evaluation can be achieved with a high quality for meeting the rapid automotive industrial inspection by means of model evaluation and estimated boundary. What is more, registration with the standard point cloud generated by the CAD model can be used to evaluate the sophisticated part that is hard to measure by the traditional method.

The rest of the paper is organized as follows. In Section 2, the laser scanning measurement system (LSMS) error analysis and the calibration process are explained. In Section 3, denoising in pre-process through various method are introduced. The measurement process and the obtained results are presented. Lastly, the conclusions of our works are given in Section 4.

## 2. Methods

### 2.1. Error Analysis

The tolerance analysis is critical for a laser scanning measurement system (LSMS) to obtain more accurate 3D point cloud information. The measuring error is inevitably caused by various impacts, but the errors in the measuring process can be calibrated by the corresponding noise-removal methods [15,16]. The errors in the LLS system mainly include environmental error, installation error, and system error. The environmental error has a small impact on the LSM system performance. Namely, the LLS can work under the temperature of 0–45 °C, at a relative humidity of 20–85%, frequency of 10–57 Hz, and vibration amplitude of 1.5 mm; thus, it can work in various industrial environments, achieving high precision.

The system errors are caused by a sensor and the corresponding moving stage. These errors can be categorized into linearity errors and random errors caused by a sensor, as well as a vibration caused by the motorized stage. The linearity errors of sensors denote the main error type in variable dimensions measuring. Besides, linearity errors depend on an operating environment. Due to the small effect of the linearity errors, these errors are considered as random errors in the measurement process. Most of the random error can be removed by the denoising process.

**Figure 1:**
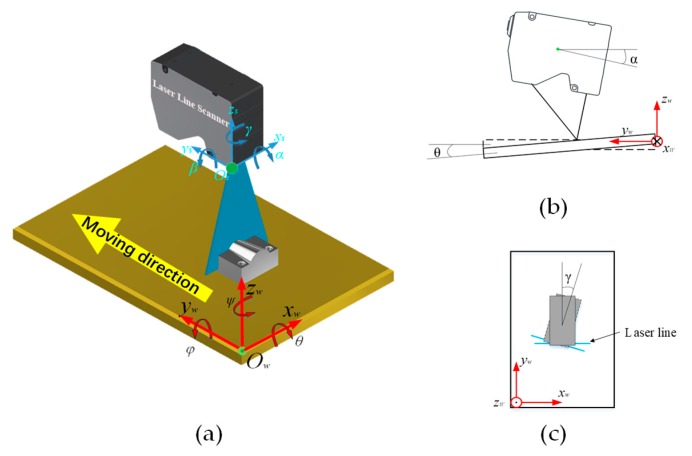
(**a**) Installation error in 3D space; (**b**) installation error in *z*-direction; (**c**) installation error in *x*-direction.

The installation error mainly refers to the error along the moving direction during the measurement process due to the deflection during installation process. The installation error is presented in Figure 1. In Figure 1, there are three axes, and the LLS can have deflections along the axes after the installation process. In order to reduce the deflection error along different axes, the calibration within a certain range should be performed, and the least squares method can be adopted.

Considering the deflection angle *α* of the LLS along the *x_s_*-axis and the worktable has deflection *θ* along the *x_w_*-axis, there are no errors in the *x*-direction; variable *L* is the install distance, and variable *Δy* is the moving distance of measurand along the *y_w_*-axis. The measurement error according to the triangular geometric relationships and sine law in the *z*-direction is given by:

(1) $$v_{z}^{x}=L(1/sin\alpha -1)\pm (\Delta y\times tan\theta [(1-cos\theta /cos(\theta \pm \alpha ))]).$$

Considering there is no deflection of the worktable, LLS has a *β* of deflection along the *y_s_*-axis. Variable g is the interval of points in the *x*-direction. The measurement error in the *x*-direction is given by:

(2) $$v_{x}^{y}=\left\lfloor \Delta ytan\beta /g\right\rfloor \times g$$

Similarly, the variable *Δx* is the distance from the center of the laser line. The measurement error in the *z*-direction is given by:

(3) $$v_{z}^{y}=L[1/cos(\beta )-1]-\Delta xtan(\beta ).$$

Considering the *γ* of deflection along the *z_s_*-axis and *ψ* of deflection along the *z_w_*-axis, the error in the *x*-direction is expressed as:

(4) $$v_{x}^{z}=\left\lfloor \Delta ytan\left(\gamma \pm \psi \right)/g\right\rfloor \times g.$$

Different dimension ceramic gauge blocks can form a ladder to calibrate the installation error. In order to acquire different plans to calibrate the LLS, we used 0.5-mm, 1-mm, and 2-mm gauge blocks in our experiment.

### 2.2. System Settings and Calibration

According to the current precision fabrication requirement, the measurement should meet the following requirements: (1) the LSMS should be able to measure workpieces with dimensions larger than 200 mm × 30 mm × 20 mm; (2) the measurement accuracy should be ±10 μm; and (3) high-speed sampling of 1 kHz should be achieved, and a maximum running speed of 50 mm/s should be obtained.

Thus, in this work, the LSMS was composed of the LLS combined with the precision motorized stage with the high-precision moving position. The sensor installed in the LSMS was LJ-V7060 from Keyence Lo, and its parameters are shown in Table 1.

A precision motorized stage is necessary, so we used a high-precision linear stage KSA400-11/X in our LSMS. The KSA400-11/X was equipped with grating scales, and it could form a closed-loop controlling mode between the motion platform and the controller, which greatly improved the control accuracy. The stroke was 400 mm, the lead of the ball screw was 5 mm, the closed-loop resolution could reach 1 μm, the maximum speed was 50 mm/s, the yawing error was less than 25′′, the pitching error was less than 40′′, the straightness of motion was less than 20 μm, and the center load was 50 kg. The manual linear stages were used to adjust the sensor to the right position to get a more precise 3D model.

**Figure 2:**
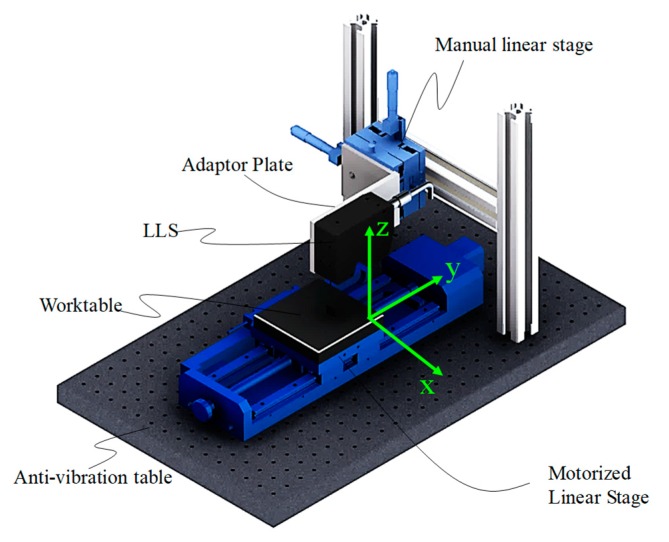
3D model of the laser scanning measurement system (LSMS). LLS is laser line scanner.

**Figure 3:**
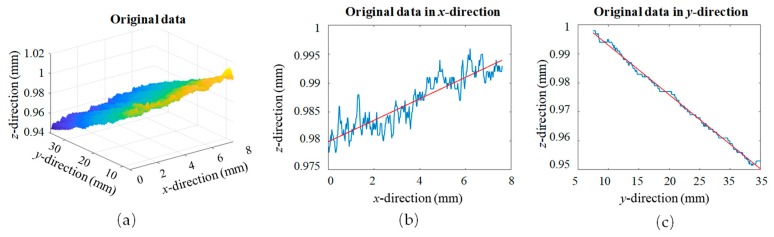
(**a**) The original data; (**b**) one of the counters in the *x*-direction; (**c**) one of the counters in the *y*-direction.

The original data of a standard gauge acquired by the constructed LSMS is shown in Figure 2. In Figure 3a, the 3D original data is displayed, and in Figure 3b,c, the contours extracted in the *x*- and *y*-directions are shown, respectively. In Figure 3a, it can be seen that there were obvious systematic errors in the measurement system, so calibration of the installation process was necessary.

The LLS measured standard ceramic gauge blocks in both *x-* and *z*-directions, analyzed each contour in these two directions that needed to be corrected separately, and fitted the angle of the contour tilt by the least square method to find the compensation coefficient of each contour. The compensation coefficient was included in the whole, and the average value was taken as the overall compensation coefficient. The contour data was first read, and the data on one of the ceramic gauge planes was intercepted for error correction. Next, the read data was read in columns, that is, the contour data intercepted in the *y*-direction for the same position point (*z*-axis direction measurement error). Then the row-reading was conducted in the direction of the light emitted by the laser triangulation sensor, which means the contour data was intercepted in the *x*-direction.

**Figure 4:**
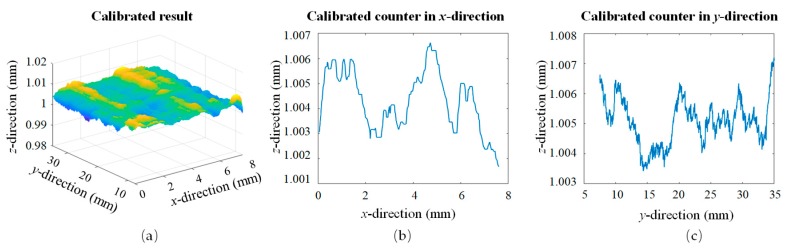
(**a**) Calibrated result; (**b**) calibrated counter in the *x*-direction; (**c**) calibrated counter in the *y*-direction.

The calibrated result is shown in Figure 4, and contours extracted in the *x*- and *y*-directions after calibration are shown in Figure 4b,c.

In conclusion, the calibration based on the least squares method applied to each profile and averaging of each coefficient factor could calibrate the tilt angle during the installation. The calibrated result was less than ±10 μm with compensation factors of 2.1 × 10^−^^3^ in the *x*-direction and −1.7 × 10^−^^3^ in the *y*-direction for errors in the *z*-direction.

## 3. Experiments and Results

### 3.1. Denoising

**Figure 5:**
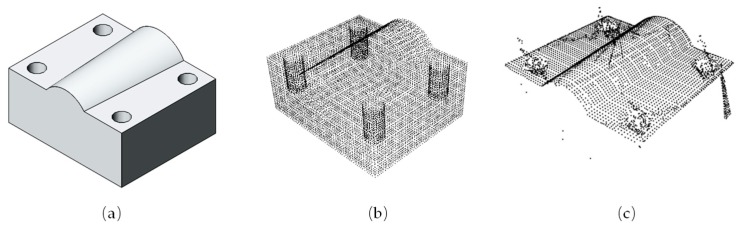
(**a**) The CAD model of measurand; (**b**) standard point cloud generated by a mesh; (**c**) scanned point cloud.

Placing the part on the stage of the calibrated measuring platform, and then performing a quick scan, the 3D point cloud information of the measurand was obtained. The storage format of 3D information mainly included common formats such as stl, step in CAD, and the geometrically defined three-dimensional format obj. The 3D information storage format commonly used in the PCL is PCD. These formats can be converted to several formats through the ASCII conversion. The part design model was meshed by 3ds Max software, and then read and used as a basis for evaluation. The 3D information of the designing part is shown in Figure 5.

The original point cloud was read from the LLS using the abovementioned system (see Figure 2) with a moving speed of 12.5 mm/s. Within 1.2 s, 960,000 points cloud could be obtained. It can be seen that there was various noise existing in the original result that would affect further data processing. Therefore, an effective denoising method was essential for achieving high measurement accuracy.

The filter used in the PCL to reduce the outrange noise was a pass-through filter, which could be used to filter the points that exceeded the measuring range of the sensor affected by the environment light. Namely, for optical measurement, a pass-through filter denoted an efficient method to remove the noise data that were out of range.

The statistical filter could effectively remove the outlier noise data that was mainly caused by the system noise, the number of noise points was sparse, and the distribution of point cloud data was discrete. The statistical filter was the most suitable for removing these kinds of errors [14,17]. The statistical filter started at a certain point in the point cloud data, and the point cloud density of the noise point determined the mean and standard deviation of the Gaussian distribution applied to the statistical filter. In this work, 100-points cloud were selected for statistical analysis. During the analysis of the neighbor points, the data that did not satisfy the Gaussian distribution were removed. The Gaussian-distribution satisfaction condition was as follows:

(5) μ−2σ≤dis(P(x,y,z))≤μ+2σ.

In Equation (5), the variable *μ* is the center of the points set and variable *σ* is the standard deviation of the points set; points that are more or less than twice *σ* are discarded, and the function *dis*() represents the calculation for distance of point.

**Figure 6:**
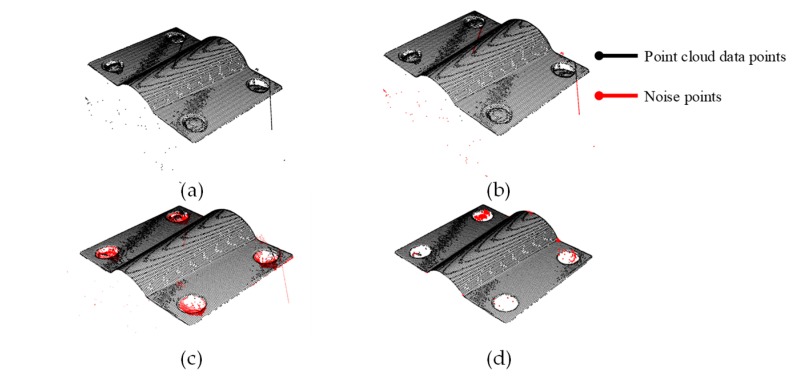
(**a**) The original point cloud; (**b**) statistical filtering result; (**c**) the random sample consensus (RANSAC) segmentation filtering result; (**d**) region growing segmentation result.

The filtering result is shown in Figure 6b. The points at the point cloud boundary were easily removed using the lower standard deviation factor, but some noise close to the model was difficult to remove by the statistical filter.

By processing via the segmentation method, the noise was effectively removed, mainly considering that the points for a specific position could be segmented by using random sample consensus (RANSAC) [18]; the coefficients of the specific region were limited, thereby the noise points were removed successfully. The RANSAC algorithm could estimate the model unaffected by noise points. Using the estimated model, some noise close to the point cloud be removed by using the coefficients, which could be especially convenient for parts in industry with known geometry. The model parameters estimated by RANSAC were used to remove the noise points by the *k*-nearest neighbors (*k*NN) algorithm searching for points that were not within the model estimation range, and limiting the noise point range [19]. Using RANSAC to evaluate the main model exit in the scanning model, we obtained the points set *Φ*(*a*, *b*, *c*, *d*). We next calculated the Euclidean distance for each point in the model to find the outliers whose distance from the model was larger than m. With the marker 0 or 1, the noise points and the signal could be classified into two different sets. The filtering result is shown in Figure 6c.

(6) $$\{\begin{array}{c} dis(P(x,y,z),\Phi (a,b,c,d))\leq m,\;\;\;\;\;\;\;\;\;\;\;\;1\\ dis(P(x,y,z),\Phi (a,b,c,d))\leq m,\;\;\;\;\;\;\;\;\;\;\;\;0 \end{array}.$$

**Figure 7:**
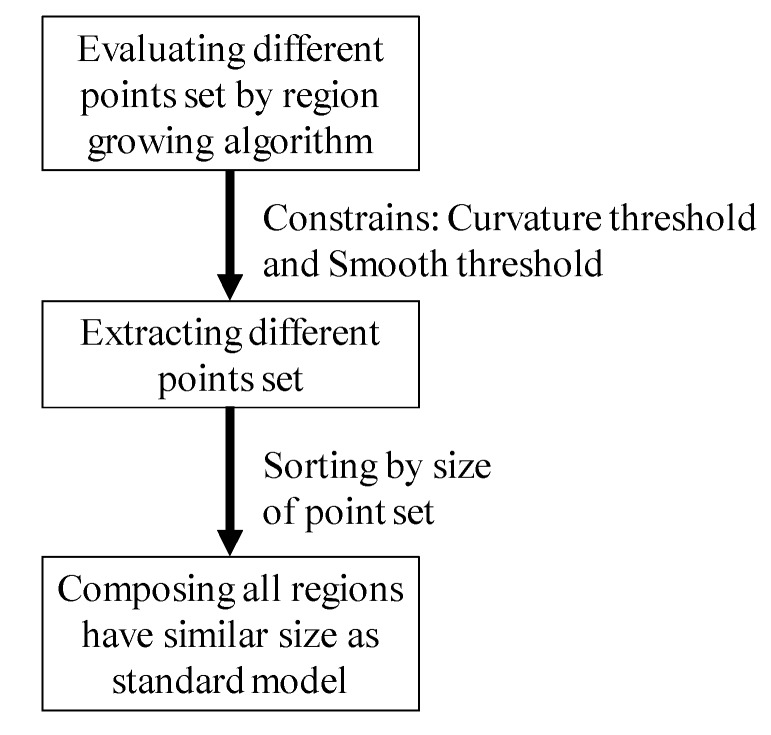
Procedures of the region growing algorithm for data denoising.

For metallic surface, there is some noise left in the sub-model when there are some holes or bosses. Spurious reflection and scattering in metallic surfaces generate some noise points close to the model [20]. The outliers caused by the internal holes were removed by the region growing algorithm [21]. Some of the noise point clouds were distributed in the area of the hole and affected by the chamfer. Few of the noise points were close to the point cloud data and difficult to remove by filters and the model segmentation method. The region growing algorithm used the local connectivity and surface smoothness as the two constraints. Thus, the noise points caused by the optical occlusion that were difficult to recognize by filters could be removed using the local connectivity. Using the region growing algorithm to estimate the points in a different region, we obtained ideal 3D information for the scanning. The filtering result is shown in Figure 6d. Additionally, the region growing algorithm was used to evaluate different point cloud sets after segmentation filtering. The procedures of denoising via region growing method are shown in Figure 7.

Besides the above-referred methods, the registration method was also used to get the point cloud matching the standard model, and then the *K*NN search algorithm was applied to each point to denoise the points far from the standard model. However, the registration result was not good enough due to the noise existence. In order to run the program faster, data compression was necessary to process the numerous data points. The voxel grid filter was used to compress the data volume through down sampling.

Different denoising methods were used for different noise points shown in Table 2. The passthrough filter was employed to remove the noise out of range. Then, the statistical filter or the RANSAC segmentation method was used to denoise the data that was unsatisfied statistically in the defined range. The noise points left in the model and close to the point cloud data were removed by the region growing algorithm.

In this section, we introduced a systematic processing for denoising and a high SNR point cloud was obtained. Except for the traditional filters employed in our first step, the segmentation method and region growing algorithm used to denoise were also introduced in our experiment. Denoising by segmentation processing combined with *k*NN only cost less 1.5 mins for 479,000 points. The efficiency of processing has greatly improved comparing with Budak’s result [10]. A metallic surface generates more noise due to the reflection and scattering on the surface. Noise left in the sub-model can be removed by the region growing algorithm to segment by constraint of local connectivity. It will cost about 6 min for 444,164 points and remove 9056 noise points in 444,164 points. The final step is still time consuming but is critical for metallic surface measurement.

### 3.2. Dimension Evaluation

The calculations of the scanned point cloud and the corresponding standard point were used for the size as well as the character location point features. First of all, the corresponding interesting area was extracted from the model, the segmentation algorithm denoted a useful tool to get interesting area, and the RANSAC denoted a powerful algorithm for recognition of some regular parts. Using the 3D plan model and cylinder model evaluation in our experiment, the main surface of the object was extracted. 

**Figure 8:**
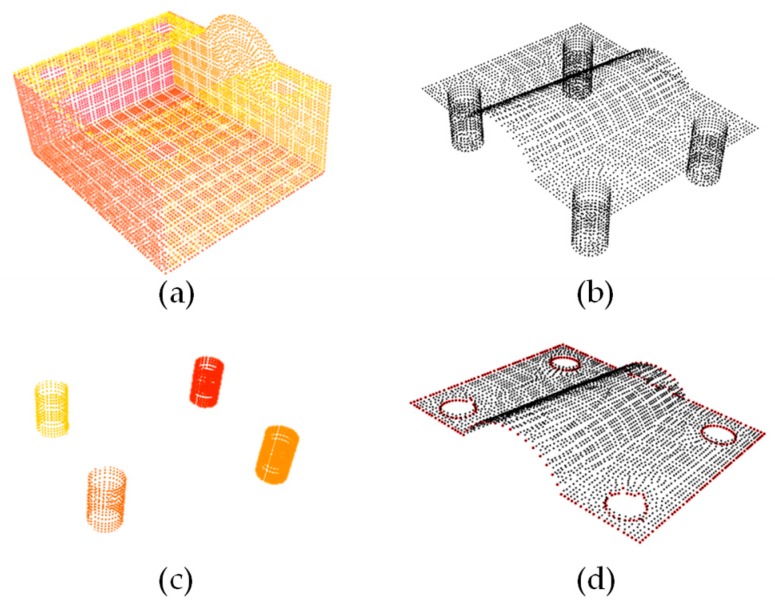
(**a**) Planes segmentation by the RANSAC; (**b**) planes extracted from the original point cloud; (**c**) cylinder segmentation by the RANSAC; (**d**) the result after the cylinders were removed, and the boundary was estimated.

In the processing, the surface of the standard point cloud was extracted, as shown in Figure 8, then the upper surface was obtained by reducing the cylinders of holes.

The normal vector of the point cloud was evaluated to estimate the boundary of the point cloud. When the boundary of the point cloud data was obtained, the position of the circular hole was estimated based on the boundary point cloud data, and the parameters of the position of the circular hole were obtained. The accuracy of the hole-positioning of the part was evaluated by comparing the corresponding center distance and hole radius with the position of the two circular holes. The main method used in the extraction of critical dimensions in the point cloud was to extract the key points of the boundary and calculate the dimension of the key points.

**Figure 9:**
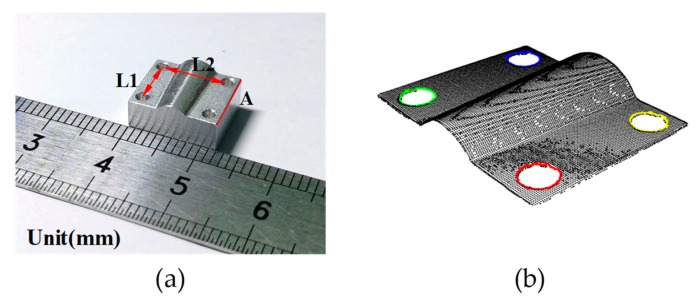
(**a**) Scanned measurand; (**b**) circle recognition with boundary estimation.

Using the key points, the dimension of the part shown in Figure 9a was estimated, and the result is given in Table 3.

In this section, we introduced a processing method for some difficult measuring dimensions that need special tools. It was easily solved in our research by point cloud data evaluation. Furthermore, no feature needs to be selected by users during processing. Most of mechanical part can be evaluated by standard geometry model. It can automatically evaluate dimensions in point cloud via boundary estimation and key points calculation. Moreover, many dimensions and features can be obtained via single measurement. Our result demonstrates that less than ±40 μm can be achieved with this method. The dimension in the *x*-direction and the radius were difficult to evaluate with high precision due to the chamfers exit. Using the results presented in Table 3, we could evaluate the dimension that was hard to measure using the traditional processing method.

### 3.3. Surface Registration

The ICP (iterative closest point) and the NDT (normal distributions transform) algorithms represent the main matching methods for 3D point clouds, but both of them have weakness in precise matching. Namely, the NDT is not as accurate as the ICP algorithm in the point cloud registration, especially regarding the rotation error [22]. When registering two different point clouds in a large different original pose, the ICP algorithm can provide a wrong pose estimation. Due to the disadvantages of the ICP, we used the ICP and its modified algorithm in our work. Since noise has a certain impact on the registration accuracy, evaluation of different registration methods is important for complex geometry part evaluation.

Comparison of different registration algorithms regarding the complexity, robustness, and precision is provided in Table 4 [23,24].

Due to the limitation of the initial matrix selection, the ICP algorithm has certain limitations. In this work, the 4-points congruent sets (4PCS) combined with the ICP algorithm, and the sample consensus initial alignment (SAC-IA) combined with the ICP algorithm were tested. The 4PCS firstly sought the original point cloud file and target point cloud file. The point cloud pair feature was obtained, and the initial transformation matrix was estimated by this feature to perform the coarse registration. Then, the fine registration was performed in combination with the ICP algorithm. The SAC algorithm was employed to estimate the normal vector of the point cloud, and the FPFH (fast point feature histograms) to calculate the original transform matrix for the ICP algorithm.

**Figure 10:**
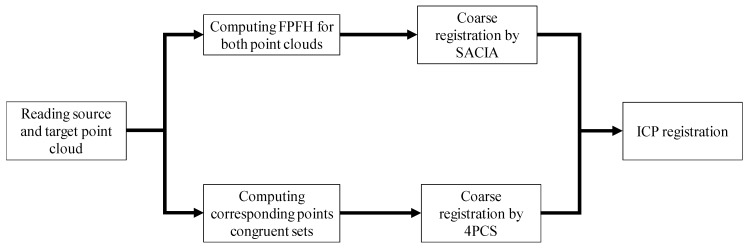
Registration steps.

**Figure 11:**
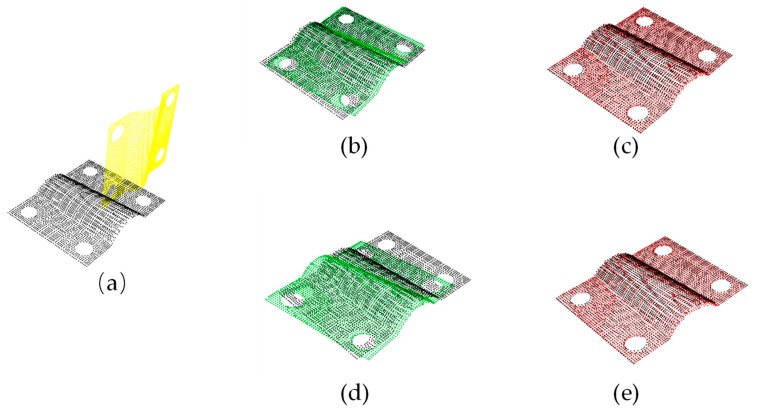
(**a**) The original point cloud data; (**b**) the coarse registration result of the 4PCS; (**c**) the fine registration by the ICP after 4PCS; (**d**) the coarse registration result of the SACIA; (**e**) the fine registration by the ICP after SACIA.

**Figure 12:**
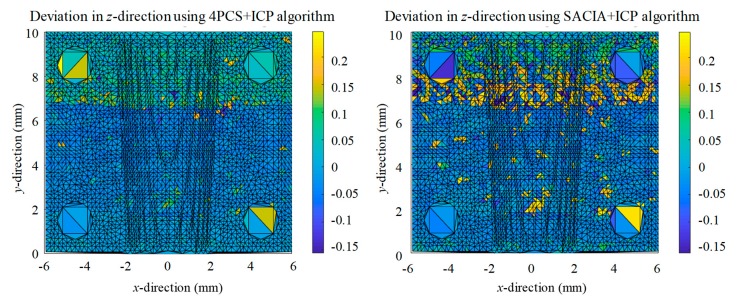
Relative deviation in the *z*-direction after the registration.

As mentioned above, the coarse registration by the SAC-IA or the 4PCs and the fine registration method combined with the ICP could provide higher precision while avoiding the inability to get a correct solution due to the limitations existing in the traditional ICP algorithm. Figure 10 shows the steps of registration in our experiment. Figure 11 illustrates the registration results. The coarse registration time consumptions for 4PCS and SACIA are 4.9 s and 114.3 s, respectively, and those for the fine registration are all 7.1 s with no more than 50 iterations, when 3969 points are processed. After registration, we evaluated these two registration methods regarding the distance in the *z*-direction; the *k*NN search algorithm was employed to find the nearest point in the plane defined by *x*-axis and *y*-axis and calculate the distance of each point pair in the *z*-direction. The relative deviation of the CAD model is shown in Figure 12.

It can be seen that the *z*-direction deviations obtained from the two registration methods were almost same. The total time consumption of the 4PCS + ICP was 12 s, about 10% of that of the SAC-IA + ICP, 121.4 s. Thus, we can conclude that the 4PCS + ICP is more effective and suitable for in-process or online industrial inspection. Generally speaking, a 12 s consumption is acceptable for a precision measurement.

**Figure 13:**
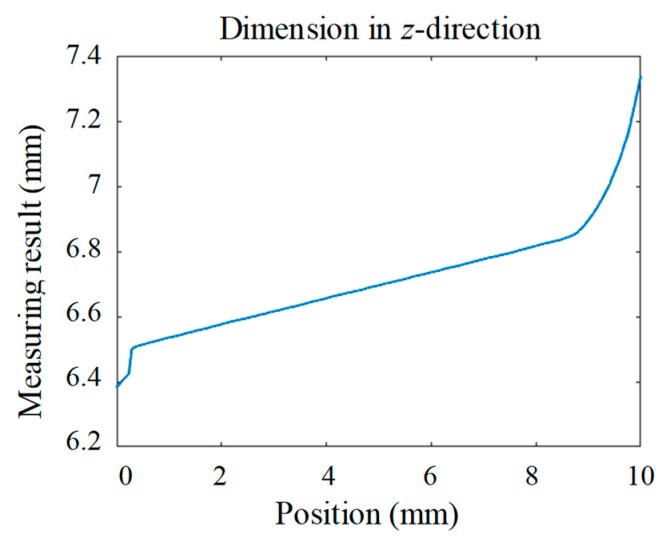
Profile result measured using the laser displacement sensor.

According to the results presented in Figure 12, the 4PCS combined with the ICP algorithm achieved more accurate results than the coarse registration by the SAC algorithm. Most of the deviation of points were within ±50 μm. The overall registration result of the 4PCS was better, and there were fewer pairs of larger-error points. Thus, in the backward of the object, there was a great deviation from the standard model for both methods, indicating where the worst part in the machining process was. The profile result measured using the high precision laser displacement sensor is shown in Figure 13.

Measuring the profile of the curved surface along the *y*-direction with the high-precision laser displacement sensor with a 0.1-μm resolution, we obtained the result shown in Figure 13. In Figure 13, it can be clearly seen that in the backward of this measured part, there was more deviation, which should be modified in the machining process. Its trend of profile demonstrates the result shown after the registration with the CAD model.

In this section, we proposed a method via registration algorithm to realize industry inspection of complex surfaces. Through comparing different point cloud via registration, one automatically can neglect the uncertainty of placing position of the measurand. It can benefit a lot for measurement of complex surfaces via large amount of point cloud data. Comparing with Kumar’s method in measurement [13], our method will be more robust on placing position for measurement of various objects. Due to 4PCS + ICP only costing 12 s for 3969 points, it can be used for online industry inspection of complex surfaces.

## 4. Conclusions

In this paper, the 3D reconstruction technology is briefly introduced, based on which the method for industrial inspection on demand of today’s intelligent manufacturing, especially for the geometrical measurement and inspection of industrial manufacturing, is proposed.

A LSMS with the LLS is designed to obtain the 3D point cloud data with high precision that is very suitable for complex environments in industrial measurement and enables high-precision 3D reconstruction of sophisticated components. The 3D point cloud data is obtained by the constructed LSMS system, which is combined with the PCL for denoising, segmentation, registration, boundary estimation, feature recognition, dimension calculation, etc. This provides a typical procedure for industrial application of 3D point cloud data processing. Using the denoising processes presented in this paper, a high SNR in the 3D information reconstruction can be obtained. Through the 3D point cloud processing, some sophisticated part that is hard to be measured directly by using the traditional measuring methods can be easily determined and used to estimate the tolerance such as circle hole location, irregular surfaces, etc.

In this paper, we have improved Igor’s method to denoise in pre-processing by adopting the *k*NN search algorithm [10]. A region growing algorithm for discarding the noise left in the sub-model was demonstrated in this research. This improved algorithm is essential for pre-processing of the noised metallic surface data and the following processing steps. Meanwhile, the 3D point cloud obtained by LSMS was employed for dimension measurement simultaneously via the method proposed in this paper, with a precision up to less than ±40 μm. What is more, we demonstrated a complex surface evaluation automatically with high efficiency by comparing the designed and scanned point clouds in which a novel registration algorithm allowing a neglect of the variation of placing position was employed.

The processing procedures based on PCL introduced in this paper show a reliable measuring performance, so it can be expected that it will experience broad market application in industry as an important method to realize the informatization in industry inspection. However, due to the large number of point clouds, processing will lose efficiency and it will cost more than 10 min when 960,000 points are processed. In our future work, we will pay attention to accelerating the algorithm to improve the efficiency and realize high-speed industry inspection.

## Figures and Tables

**Table 1:** Parameters of the LJ-V7060.

Parameters		LJ-V7060
Reference install distance		60 mm
Measurement range	*z*-axis	±8 mm
	*x*-axis	15 mm
Output power		10 mW
Wavelength		405 nm
Linearity in the *z*-direction		±0.1% of F.S.
Profile data interval along the *x*-axis		20 μm

**Table 2:** Comparison of different noise reduction methods.

	Denoising Method	Statistical Filtering	Segmentation with SAC	Segmentation with Region Growing
Point Position				
Away from point cloud		√	√	√
Closed to point cloud		-	√	√
Noise points left in the model		-	-	√

**Table 3:** Measurement results obtained by the 3D information processing.

Dimension (mm)	Micrometer/Caliper * (mm)	Evaluation via Point Cloud (mm)	Deviation (mm)
A (10)	9.989	10.025	0.036
L1 (7)	7.02 *	7.06	0.04
L2 (9)	9.08 *	9.12	0.04

* Using caliper for the measurement result.

**Table 4:** Comparison of different registration methods.

Algorithm	Complexity	Accuracy	Robustness
ICP	Low	High	Low
4PCS + ICP	Medium	High	Medium
SAC-IA + ICP	High	High	High
